# Experimental manipulation of ecological constraints influences conflict but not cooperation in the humbug damselfish *Dascyllus aruanus*

**DOI:** 10.1098/rspb.2025.3229

**Published:** 2026-10-14

**Authors:** Rebecca Branconi, Marian Y. L. Wong, Daniel B. Stouffer, Theresa Rueger, Peter Buston

**Affiliations:** ^1^ Zaratan Marine Research & Higher Education Centre, World Biodiversity Association, Sezione Arcipelago Toscano, Portoferraio, 57037, Italy; ^2^ Department of Biology, The Pennsylvania State University, University Park, PA, USA; ^3^ Department of Biology, Boston University, Boston, MA, USA; ^4^ School of Science, University of Wollongong, Wollongong, New South Wales, Australia; ^5^ Department of Evolutionary and Integrative Ecology, Leibniz Institute of Freshwater Ecology and Inland Fisheries (IGB), Berlin, Germany; ^6^ Dove Marine Laboratory, School of Natural and Environmental Sciences, Newcastle University, Newcastle Upon Tyne, UK

**Keywords:** animal societies, social fish, social evolution, habitat dependence, coral reefs, social constraints

## Abstract

Animal societies vary widely in levels of cooperation and conflict, and understanding the causes of this variation is a central goal in evolutionary biology. As with other features of animal societies, relatedness, competitiveness and ecological constraints (e.g. habitat saturation and risks of dispersal) have been proposed as key factors explaining variation in cooperation and conflict. However, the exact causes remain unclear. We used the humbug damselfish (*Dascyllus aruanus*)—a small coral reef fish that occupies coral heads—to experimentally test the prediction from Emlen's ecological constraints model that relaxed constraints increase conflict and decrease cooperation within social groups. We manipulated ecological constraints by providing a vacant coral near groups and examined changes in social interactions. We also assessed how variation in social context (e.g. individual size, group size and size ratios) and ecological context (e.g. territory size, habitat availability and local group density) modulated these effects. Relaxing ecological constraints led to increased conflict in groups occupying larger corals, in areas with greater habitat availability and in larger groups, whereas it led to decreased conflict by larger individuals and in areas with higher local group density. No effect on cooperative behaviours was observed. These findings suggest that ecological constraints influence conflict within groups, but that this relationship is context-dependent and modulated by both ecological and social factors.

## 1. Introduction

Understanding the causes of inter- and intraspecific variation in the structure and organization of animal societies is a major goal of evolutionary biology. Societies can vary greatly in their reproductive partitioning, levels of cooperation and conflict, group size, structure and stability. It is widely appreciated that reproductive partitioning and group stability are dependent on a combination of genetic, social and ecological factors [1–3]. For example, subordinate individuals are more likely to forgo their own reproduction and remain within a group when relatedness is high, there are strong competitive asymmetries, and there are constraints on independent breeding (e.g. [4–9]). These same three factors—relatedness, competitive asymmetry and ecological constraints—are likely to influence levels of cooperation and conflict within groups, but exactly how and why they do so in nature remains unresolved.

Ecological constraints encompass both habitat saturation, referring to the availability of suitable habitat patches and the degree to which such patches are occupied by competitors, and risks of dispersal, referring to the costs involved in moving and finding suitable patches of habitat in which to reside and reproduce [1,10–13]. The relaxation of such ecological constraints, which can occur through decreasing habitat saturation and/or risks of dispersal, affects the costs and benefits of group formation and maintenance, and has been shown to influence individual dispersal decisions and group stability under both natural and experimental conditions (e.g. [4–9,14–16]). As such, an experimental relaxation of ecological constraints could potentially also affect within-group cooperation and conflict prior to group dissolution (e.g. [17–23]).

Multiple theoretical models have been developed to predict how variation in ecological constraints influences cooperation and conflict within social groups. Early models suggest that conflict will be more likely when there are weak ecological constraints, and cooperative behaviours will be more likely when there are strong ecological constraints [1]. Transactional models of reproductive skew, in which group members yield shares of reproduction to each other in return for cooperation, also predict that groups will be more prone to conflict when ecological constraints are weak [24–27]. Furthermore, a model that synthesizes the ecological-constraints hypothesis, the life-history hypothesis and the benefits-of-philopatry hypothesis suggests that when there is a high likelihood of territory inheritance, which often co-occurs with strong ecological constraints, there will be greater cooperation within groups [28]. Also, the dual benefits framework model suggests that cooperation will be promoted and driven by resource defence benefits, in social groups in which ecological constraints limit independent breeding [29]. On the other hand, tug-of-war models of reproductive skew, in which group members invest group resources in a tug-of-war over their respective reproductive shares, suggest that the degree of within-group conflict will increase as ecological constraints on solitary breeding increase (i.e. the non-cooperative options become less attractive) [30]. Despite this large number of models, however, few observational studies have tested how ecological constraints affect cooperation and conflict in social groups (e.g. [17,21,22]), and even fewer experimental studies have investigated the effects of manipulating ecological constraints on cooperation and conflict within social groups [4,18–20,23]. Importantly, these studies suggest that there is not a universal response of animal social groups to different ecological constraints. Further, the effects of an experimental manipulation, if any, can vary across species and between types of ecological constraints. In addition, other subtle factors (e.g. variation in relatedness) can also modulate the relationship between ecological constraints and individual behaviour. Here, by experimentally testing the predictions of Emlen's original model [1], we focus on the role of ecological constraints in determining levels of cooperation and conflict within social groups of a coral reef fish.

In the last two decades, Indo-Pacific coral reef fishes, such as *Amphiprion percula* and *Paragobiodon xanthosoma*, have served as key model systems for studying the evolution of cooperative behaviour in marine environments [31–33]. These species are characterized by low mobility, high reproductive skew and relatively simple social systems, and studies on them have shown that strong ecological constraints—such as habitat saturation and risks of movement—play a central role in shaping social structure and dispersal decisions [14,16,34–36]. However, their relatively simple social organization limits our ability to understand how ecological constraints influence more complex societies. To address this, we focus on the humbug damselfish, *Dascyllus aruanus*, a species with higher mobility, lower and more variable reproductive skew and more complex social organization [14,16].

*Dascyllus aruanus* is widespread throughout the Indo-Pacific region, living in social groups in close association with colonies of branching corals [37,38]. Within each discrete coral patch there is a single group of mostly non-relatives [38,39] where the largest individual(s) is male (are males), the intermediate individuals are females and the smallest individuals are non-breeders [40–43]. The number of individuals in the group increases as coral size increases and, = in contrast to *A. percula* and *P. xanthosoma* that are strictly monogamous, the mating system of *D. aruanus* shifts from monogamy to polygyny to polygynandry with increasing group size [42,44]. Additionally, unlike *A. percula* and *P. xanthosoma*, there is a random distribution of size ratios between individuals of adjacent ranks in *D. aruanus* [45] and the reproductive skew is highly variable—both small and large individuals can reproduce, although larger individuals generally attain a larger reproductive share than smaller ones [42]. Interestingly, *D. aruanus* individuals sometimes have multiple coral hosts within their territories, and fish move between them, both on their own and in groups [46]. However, the movement of individuals between different groups and territories is limited for three reasons [47]: (i) suitable habitat patches are discrete, (ii) it is risky to move between patches of suitable habitat [48] and (iii) residents of occupied habitat actively repel unfamiliar conspecifics [49,50]. Therefore, *D. aruanus* experiences naturally occurring variation in ecological constraints, making it the perfect candidate to experimentally investigate how ecological constraints influence the stability of complex animal societies. Within these groups, aggressive interactions (e.g. chases and displays) are frequent, and we consider these interactions to be indicative of conflict (table 1) [51]. By contrast, cooperative interactions (e.g. submissive and affiliative displays) are less frequent and we consider these interactions to be indicative of peaceful cooperation, because by reducing conflict they will increase the fitness of both the actor and the recipient (table 1) [33,51–53].

**Table 1.** Summary of the social behaviours—conflictive and cooperative interactions—used in this study. Conflictive interactions are considered those that involve potential or actual harm to another individual within the group. Peacefully cooperative interactions are considered those that promote conflict resolution and reduce costs of conflict between group members (from [51]).

behavioural category	description
**conflictive interactions between group members**	*aggressive behaviours*: chasing (focal fish quickly swims/darts towards another fish) biting (focal fish bites another fish) aggressive display (focal fish raise all fins and swim upwards to another fish and then takes an oblique position and quivers and finally swims in parallel) ram (focal fish makes contact with another fish using the head or mouth region, but no obvious bite is taken; jaws remain closed)
**peacefully cooperative interactions between group members**	*submissive behaviours*: flee (focal fish quickly swims away from another fish as a response to received aggressive behaviours) hiding (focal fish quickly swims inside coral branches or holes into the rocks to place itself out of another fish's view as a response to received aggressive behaviour) submissive display (the head of the focal fish is directed downwards, sometimes entirely vertical, the tail is upwards and the whole body trembles) head down (the head of the focal fish is directed downwards, sometimes entirely vertical, the tail is upwards) *affiliative behaviours*: following a group member (focal fish follows another one within the group) meeting a group member (focal fish meets another one and swim together)

This study investigates whether cooperation and conflict within groups of the humbug damselfish *D. aruanus* are influenced by a manipulation of ecological constraints. Based on the expectations of Emlen's original model [1] and on the results of previous experimental studies in other species, we predict that conflictive interactions will be more likely and peacefully cooperative interactions less likely when ecological constraints are experimentally relaxed. Indeed, we find that the relaxation of ecological constraints results in changes in the levels of conflict within social groups, but not cooperation and the results are context-specific. These results provide new experimental insights into the factors influencing social interactions within social groups.

## 2. Methods

### (a) Study population

For this study, we used a population of *D. aruanus* in Lizard Island Lagoon on the northern Great Barrier Reef, Australia (14°40′ S, 145° 27′ E). The study was conducted from January to March 2016 at three different diving sites: north of Palfrey Island, Bird Islet and Trawler Beach. All fieldwork was conducted using SCUBA at depths less than 20 m. Within the dive sites, the reef consisted of areas of rubble and living corals interspersed with extensive areas of sand. In total, we haphazardly located and mapped 82 host corals using GPS, from which we selected 18 discrete groups with a minimum of seven individuals. Counts of resident fish were carried out multiple times on different days to ensure an accurate estimate of group size in the focal coral and minimize errors related to (i) movements of individuals between different coral hosts within their territory, or (ii) considering transient fish as actual residents. Only groups that showed the same number of individuals across different days were used in our sample (*n* = 18 groups).

### (b) Fish collection and tagging

Fish from each group were anesthetized using a clove oil solution [54], captured using a slurp gun or hand net, and placed into zip-lock bags to be transferred to the boat. Once on board, bags were inflated with air and placed in an insulated storage box to protect the fish from direct sunlight. Fish were transported to the laboratory facilities at Lizard Island Research Station within 1 hour of capture. Each fish was uniquely tagged using topical surgical glue and coloured plastic films that were easily detectable by video/photo cameras [55]. After tagging, fish were held under observation to confirm normal behaviour and health status before being returned to their original site the following day and released onto their home focal coral. No signs of stress were observed after tagging and within 5 minutes of the procedure, the behaviour of marked individuals had returned to normal (i.e. regular breathing rate, movement and buoyancy). Following reintroduction, individuals appeared to resume normal behaviour within 15 minutes. Ecological measurements were collected immediately after fish reintroduction to their home corals, and experimental video recordings (with and without a vacant coral treatment) were initiated 1 hour after reintroduction to allow sufficient time for acclimation and to minimize disturbance associated with handling and diver activity. During periods when groups were temporarily removed from their corals for tagging, empty corals were occasionally visited by other fish species; however, no occupation was observed at any point during the study. The haphazard selection of more ‘discrete’ groups minimized the likelihood of such movements.

### (c) Experimental design and data collection

#### (i) Characterizing social context

To control for natural variation in social context on social behaviour, we characterized the social context of the 18 focal groups (figure 1a). First, to determine individual size, the standard length (SL) of each fish, the straight-line distance from the tip of the snout to the base of the caudal fin [56], was measured to 0.1 mm using calipers. Next, to determine relative competitive ability, the size ratio of an interacting pair (‘actor/receiver size ratio’) was estimated as the SL of the actor divided by the SL of the receiver. Finally, group size was determined by counting the number of resident fish on the focal host coral (*n* = 18 groups, *n* = 204 individuals, mean number of individuals per group ± s.d. = 11.3 ± 4.5).

**Figure 1.**
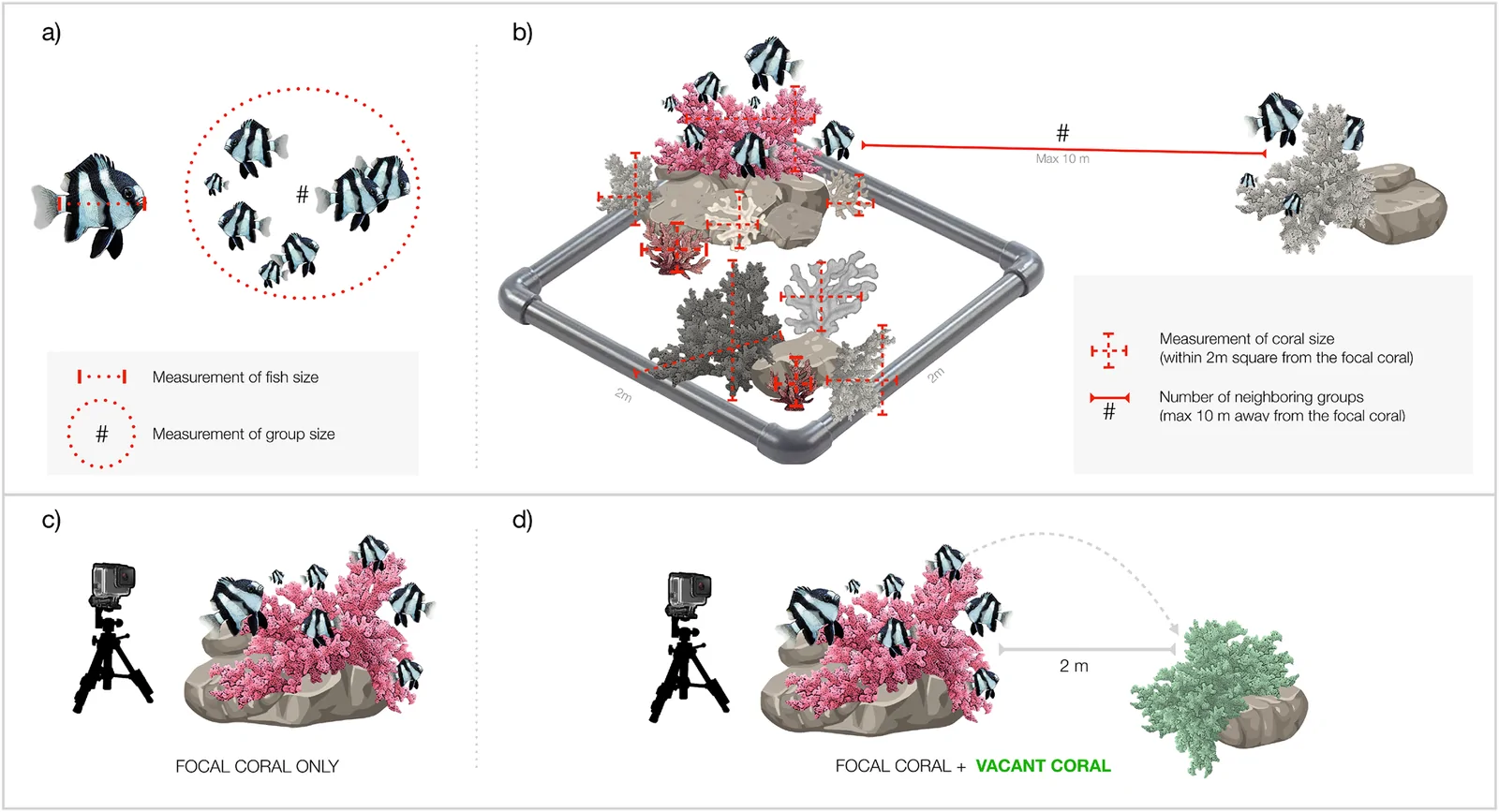
Schematic of the data collection and experimental design: (a) characterization of the social context, i.e. individual standard length, size ratio and group size, (b) characterization of the ecological context, i.e. ecological volume of each focal coral (in pink), ecological volume of the neighbouring branching corals (in grey) within 2 m^2^ and number of neighbouring groups present on other corals within 10 m radius and (c,d) manipulation of the ecological constraints by the addition of a vacant coral (in green) placed 2 m away from the focal coral (in pink); the collection of the behavioural data was performed through the analysis of video recording and the order of the videos of the groups recorded with and without the vacant coral was randomized.

#### (ii) Characterizing ecological context

To control for natural variation in ecological context on social behaviour, we characterized the ecological context of the 18 focal groups. First, to estimate the territory size of each group, the size of each focal coral was determined by measuring the length, width and height of the coral colony using a measuring tape and calculating the ecological volume [57]. Then, to estimate immediate habitat availability, the ecological volume of each neighbouring branching coral within a 2 m^2^ of the focal coral was measured and summed (figure 1b) [38]. Finally, to estimate local habitat density, the number of neighbouring groups of *D. aruanus* was counted within a 10 m radius of the focal coral (figure 1b). Pearson's correlation determined that there was no linear correlation between immediate habitat availability and local density (*r*(16) = 0.23, *p* = 0.36).

#### (iii) Experimental protocol

After characterizing the social and ecological context of the focal groups, we chose vacant corals that would be used to experimentally relax ecological constraints. For the selection of the vacant coral, we surveyed the coral population and identified a small number of corals that were healthy and movable (i.e. attached to small rocks). One of these corals (species: *Seriatopora caliendrum*; ecological volume: 16880.64 cm^3^) was collected and used as the vacant coral for all experiments—corals of this size and species were regularly used by *D. aruanus*. At the end of each pair of experimental recordings, the vacant coral was removed from the area and placed in a designated low-disturbance holding area for use with the next group on the following day. This coral remained healthy throughout the entire experiment. A single coral head was used as the standardized vacant ‘outside option’ to control for variation in coral characteristics and ensure consistency across trials. Additionally, the number of suitable corals that could be reliably manipulated at depths of up to 20 m was limited and disturbance to the local coral community was minimized by restricting manipulation to a single colony. This design limits replication of the outside option, and its implications are addressed in the Discussion.

Having sourced the vacant coral, we used GoPro Hero 3 and Hero 3+ (www.gopro.com) cameras fixed on tripods to record each focal group twice (figure 1c,d). For half the groups, the recordings were first conducted without the vacant coral and then immediately afterwards with the vacant coral present. For the other half, this order was reversed so that they were recorded with the vacant coral present first and then without the vacant coral second. This controlled for any effects of order of vacant coral presentation on behaviour. Each recording lasted for a duration of 34 minutes, between 10:00 and 16:00 hours. The GoPro camera was placed 1 m away from the group. Video recordings began 1 hour after each group was released into their focal coral to allow for acclimation of the fish following the collection and tagging procedures.

All videos were scored by the same observer (R.B.) using the program VLC Media Player 2.1.3 (www.vlc-media-player.en.uptodown.com). Video scoring commenced 4 minutes after the start of the recording to account for the time necessary for the scuba diver to leave the area, resulting in 30 minutes of total scoring time. Social behaviours (conflictive and cooperative interactions, table 1) were scored along with the identities of the individuals performing (i.e. actor) and receiving (i.e. receiver) the behaviours. Each individual within each group was scored, and only one individual was scored at a time to ensure the accuracy of behavioural data collection, resulting in approximately 4320 hours of video analysis over a 2-year period.

To maximize the amount of data for analyses, we quantified the time between behavioural events. Specifically, the time(s) between each successive conflictive interaction (e.g. bite and chase) and between each successive cooperative interaction (e.g. submission or affiliation) were calculated for each individual over the entire time period it was observed. For example, if an individual chased another at 5 m 30 s and then performed a bite at 5 m 45 s, the time between these conflictive behavioural events was scored as 15 s. To address instances where time intervals were recorded as zero, a small adjustment constant, commonly referred to as a ‘fudge factor’, was added. This modification ensured numerical stability in subsequent analyses and prevented computational issues, such as the misrepresentation of seemingly instantaneous events. Time between behavioural interactions is synonymous with the frequency of a behaviour occurring but is more informative than count data (for further justification of the use of time differences as the basis of the analysis, see [58,59]).

#### (iv) Statistical analysis

Behavioural data were analysed using mixed model analysis with the *glmmTMB* package [60] in R [61]. To determine whether the intensity of social behaviours varied with respect to ecological constraints after controlling for ecological and social context, we used three generalized linear mixed-effect models (GLMM) with a gamma error term (*log link*), where the dependent variables were the time differences between: (i) conflictive interactions, (ii) cooperative (affiliative) interactions, and (iii) cooperative (submissive) interactions, respectively. For cooperative interactions, submissive and affiliative behaviours were modelled separately, given that these two types of behaviours are likely to be exhibited under different social contexts. Importantly, a decrease in the response variable ‘time difference between interactions’ (seconds) indicated an overall *increase* in the intensity of cooperative or conflictive interactions, whereas an increase in time difference indicated a *decrease* in the overall intensity of these interactions. The primary independent variable was treatment (‘focal coral only’ versus ‘focal coral plus vacant coral’, categorical). To investigate potential effects of social context on social behaviours, we also included three social predictors: (i) actor size (numeric and scaled), (ii) actor/receiver size ratio (numeric and scaled), and iii) group size (count and scaled) as fixed effects in the models and as two-way interactions with treatment. To investigate potential effects of ecological context on social behaviours, we further included three ecological predictors: (i) the ecological volume of the focal coral (numeric and scaled), (ii) the total ecological volume of neighbouring corals (numeric and scaled), and (iii) the number of neighbouring groups (count and scaled) as fixed effects in the models and as two-way interactions with treatment. Scaled predictor values were used to improve model convergence. To control for the number of individuals with which any fish can interact, group size minus one (since there are no self-interactions in this context) was also included as an offset in the models (i.e. *log (group size − 1)* to match the *log* link function). Group ID, actor ID and receiver ID (crossed random intercepts) were also included in the models to control for the lack of independence between multiple measures from the same fish and same group. Significance tests for GLMMs were performed by likelihood ratio tests of the full model with the effect in question against the model without the effect. No homoscedasticity or obvious deviations from normality were detected by visually inspecting the residual plots; no outliers or high variance inflation factors were detected in any of the best-fit models by testing for outliers and collinearity between variables with *performance* [62]. Conditional and marginal *R*^2^ were calculated using Nakagawa's *R*^2^ in *performance* [63–65]. For some models, the random term variance component was zero and conditional *R*^2^ could not be calculated. This had no effect on the model components reported here [66]. In all cases, the full model with all hypothesized main effects and interaction effects is reported.

## 3. Results

### (a) Behavioural sequence following experimental manipulation

Following tagging and reintroduction, fish showed rapid recovery of normal behaviour, with no observable differences in activity compared with pre-handling conditions. Individuals typically resumed normal swimming and positioning within the coral within approximately 5 minutes after release. Upon return to their focal coral, most individuals initially remained sheltered within the coral structure for 10–15 minutes, before resuming typical group activity.

Exploration of the vacant coral began shortly after its presentation, with the first visits made by the largest individuals within 11 minutes of the experimental setup. Visits were generally brief and were performed by both single individuals and multiple group members. Across trials, individuals visited the vacant corals on an average of 6.00 times and spent on average 5.31 ± 3.18 minutes inspecting the vacant coral out of 30 minutes of video, with 22% of groups showing at least one exploratory visit. No sustained occupation of the vacant coral was observed in any trial, and individuals consistently returned to their original focal coral following exploration, i.e. no group dissolution occurred.

### (b) Effects of ecological constraints on conflictive interactions

Our results show that there was a main effect of treatment (i.e. whether there is a vacant coral present or not) on the time between conflictive interactions, i.e. the intensity of conflict (*χ*^2^_(1)_ = 13.5, *p* < 0.001), and a main effect of actor/receiver size ratio (i.e. the relative size of actor versus receiver) on the time between conflictive interactions (*χ*^2^_(1)_ = 4.3, *p* = 0.04). All other factors (i.e. actor's size, group size, the ecological volume of the focal coral, the total ecological volume of neighbouring coral and the number of neighbouring groups) were not significant predictors of time between conflictive interactions (supplementary material, table S1). Importantly, our results show significant interactions between the experimental relaxation of ecological constraints and various ecological and social factors (figure 2a–f; supplementary material, table S1), which will be discussed below.

**Figure 2.**
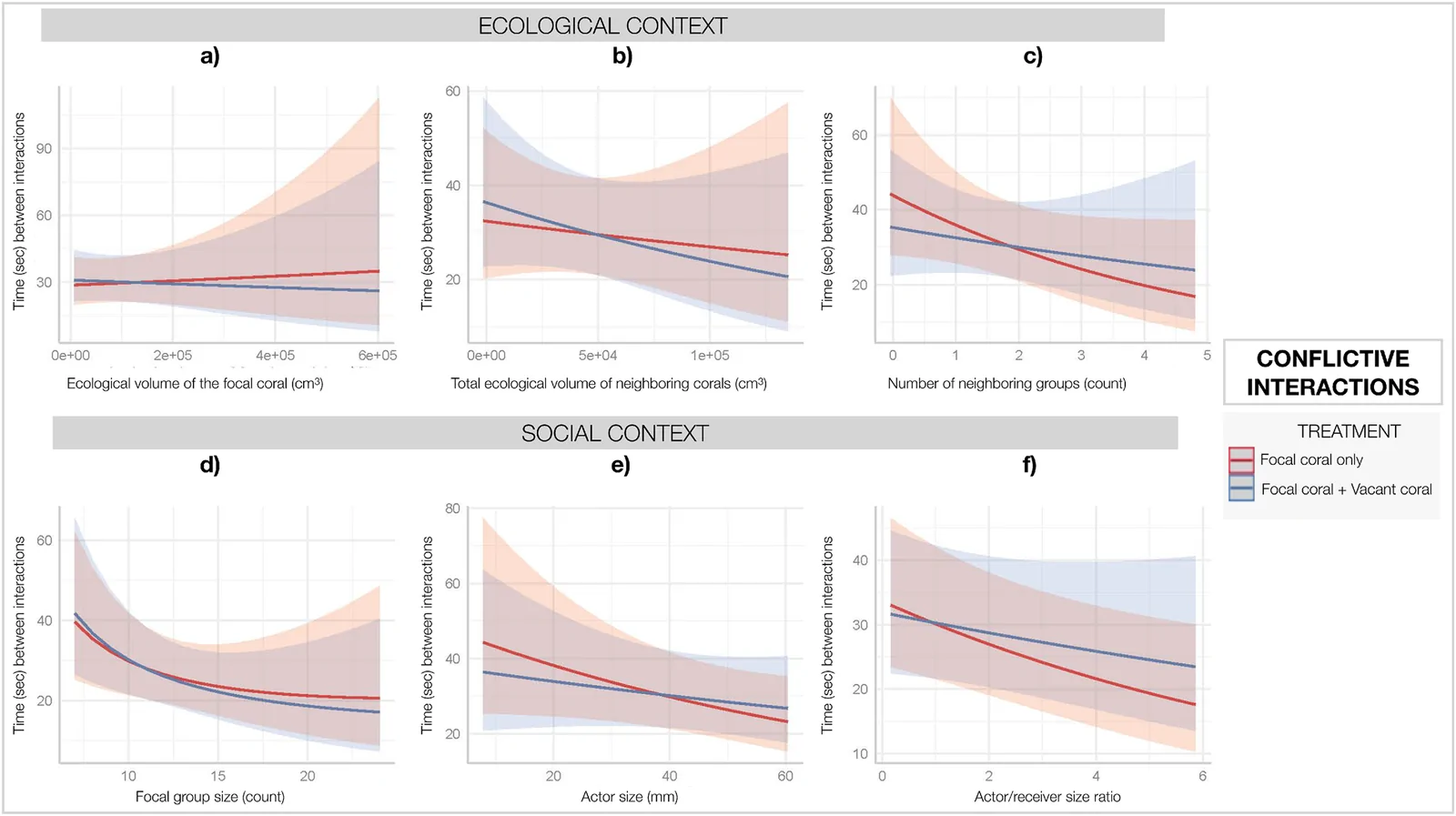
Comparison of the effects of ecological (a–c) and social (d–f) context on time difference between conflictive interactions in *D. aruanus* under natural (red) and experimental (blue) treatments; (a) effect of treatment by ecological volume (cm^3^) of the focal coral, (b) effect of treatment by total ecological volume of the neighbouring corals (cm^3^), (c) effect of treatment by number of neighbouring groups, (d) effect of treatment by focal group size, (e) effect of treatment by actor size and (f) effect of treatment by actor/receiver size ratio. Lines represent estimated best fits from models and shading represents 95% confidence intervals. In all panels, non-focal predictor variables are set to their average value across the data set.

Regarding the ecological context (figure 2a–c), the frequency of conflictive interactions was dependent on the interaction between experimental treatment and focal coral ecological volume (*χ*^2^_(1)_ = 25.6, *p* < 0.001), ecological volume of the neighbouring corals (*χ*_(1)_ = 23.3, *p* < 0.001) and number of neighbouring groups (*χ*^2^_(1)_ = 88.9, *p* < 0.001). Specifically, the slope of the relationship between volume of the focal coral and time difference between conflictive interactions (figure 2a) and the slope of the relationship between volume of the neighbouring corals and time difference between conflictive interactions (figure 2b) were more negative when a vacant coral was present compared with when absent. By contrast, the slope of the relationship between the number of neighbouring groups and time difference between conflictive interactions (figure 2c) was less negative when a vacant coral was present.

Regarding the social context (figure 2d–f; supplementary material, table S1), the intensity of conflictive interactions was dependent on the interaction between the experimental treatment and focal group size (*χ*^2^_(1)_ = 20.9, *p* < 0.001) (figure 2d) and actor size (*χ*^2^_(1)_ = 10.0, *p* = 0.002) (figure 2e). More specifically, the slope of the relationship between group size and time difference between conflictive interactions (figure 2d) was more negative when a vacant coral was present. By contrast, the slope of the relationship between actor size and time difference between conflictive interactions (figure 2e) was less negative when a vacant coral was present. Finally, there was a significant main effect of actor-receiver size ratio on conflictive interactions (*χ*^2^_(1)_ = 4.3, *p* = 0.04), demonstrating that conflictive interactions became more frequent as actors became larger than receivers, regardless of the degree of ecological constraints (figure 2f).

### (c) Effects of ecological constraints on peacefully cooperative interactions

By contrast to conflictive interactions, our results show that there was no effect of treatment (i.e. whether there is a vacant coral present) on the intensity of peacefully cooperative interactions (supplementary material, table S2). Importantly, the effect of the experimental relaxation of ecological constraints on both submissive and affiliative behaviours did not depend on the current ecological context of groups and individuals or on the social context (supplementary material, table S2a.1), i.e. there were no statistically significant two-way interactions. The lack of significant treatment and interaction effects cannot be attributed to over-parameterization in the full models, as these effects remained non-significant in simplified models without two-way interaction terms (supplementary material, table S2b.1). Overall, fish in larger groups showed more submissive behaviours (*χ*^2^_(1)_ = 22.3, *p* < 0.001), but there were no significant effects of any other predictors on the intensity of submissive or affiliative behaviours.

## 4. Discussion

To test the hypothesis that cooperation and conflict would be influenced by ecological constraints, we investigated the effect of a manipulation of ecological constraints on the intensity of conflictive and peacefully cooperative interactions (affiliation and submission, which were tested separately) in the humbug damselfish *D. aruanus*. We show that the effect of the manipulation of ecological constraints on conflictive interactions depends on both ecological and social context. By contrast, we show that there is no effect of the manipulation of ecological constraints on peacefully cooperative interactions—neither affiliation nor submission. Overall, the addition of a vacant coral modulated the overall relationships between various social and ecological factors and the intensity of conflict (but not cooperative interactions).

A possible limitation of our design was the use of a single coral head as the vacant ‘outside option’, which restricts replication at the habitat level and may limit the generality of our findings. The attractiveness or quality of this coral (e.g. in terms of size or structural complexity) could have influenced dispersal incentives and, consequently, the intensity of interactions observed within groups. However, by standardizing the outside option across trials, we minimized variation in habitat quality and were able to isolate the effects of experimentally relaxed ecological constraints on social interactions. Future studies incorporating multiple vacant corals of varying quality would help determine how habitat heterogeneity shapes these dynamics.

### (a) Conflict and ecological constraints

In general, the ecological constraints hypothesis predicts that conflict will increase in groups as ecological constraints are relaxed, and the magnitude of the effect will be modulated by individual traits, social context and ecological context [1]. The basic rationale for this is that, as outside options improve, individuals will become more demanding of resources within the group and conflict will increase, up until the point that all individual demands cannot be met and the group dissolves via forcible evictions or voluntary departures [24,25]. Our results suggest that, in the presence of a vacant coral, the intensity of conflict increased in larger focal corals, when neighbouring corals were larger and when in bigger groups. On the other hand, when a vacant coral was available, the intensity of conflict decreased when many neighbouring corals were present, contrary to the original hypothesis. Below we highlight several possible mechanisms that could help us better understand these ecological results.

Adding an empty coral close to a larger focal coral (or closer to larger neighbouring corals) is likely to increase the attractiveness of an individual's outside options, as well as an increased possibility for them to safely leave the group. This, in turn, could lead to increased conflict between group members, as subordinates demand more resources from dominants and dominants consequently have to suppress them. Similarly, when the focal group is large, there is increased competition over reproduction and resources between the many group members. Therefore, the addition of a vacant coral becomes an attractive option for subordinates in larger groups, leading to increased resource demands by the subordinates and hence conflict within the current focal group. Anecdotal support for this explanation comes from our experimental manipulation, where some individuals (typically the larger ones) moved between the focal coral and the vacant coral, suggesting that relaxing ecological constraints could induce temporary group dissolution. Larger sexually mature individuals might move more often than small ones since large individuals are less likely to be preyed upon while moving between groups [67] and/or because they have reduced probabilities of rank ascension within their current groups as there are fewer individuals ahead of them to die [68].

Our results also demonstrate that the presence of a vacant coral led to a reduction in conflict when there were many neighbouring groups, contrary to Emlen's original hypothesis. One possible explanation for these results could relate to the balance of intra versus intergroup conflict. In the presence of many neighbouring groups, saturation of the surrounding environment is greater, and consequently subordinates have fewer options to disperse and breed independently. Even if they did disperse, they are likely to be repelled from settling in neighbouring groups by residents [49,50]. Therefore, a vacant coral in the vicinity of a highly saturated environment may be a less attractive option for subordinates, given increased competition with individuals from the other neighbouring groups over this empty coral resource. Consequently, subordinates may become less aggressive towards their fellow group members in order to be tolerated within the group. In addition, the presence of many neighbouring groups can pose a threat to the resources of an established group, incentivizing elevated between-group conflict over within-group conflict [69–71]. Indeed, theoretical work predicts that conflict with outsiders should affect within-group dynamics [72,73], and empirical studies have already demonstrated how interactions with out-group rivals, as well as simulated out-group territorial intrusions, deeply affect within-group social dynamics [69,74,75]. Therefore, future studies investigating the balance between inter- and intra-group dynamics under conditions of relaxed ecological constraints would be important to better understand the association between these factors and their effect on within-group social dynamics in *D. aruanus*.

When considering body size effects, our results suggest that larger and smaller actors behaved differently depending on the availability of a vacant coral. When a vacant coral was available, larger actors were less aggressive whereas smaller actors were more aggressive. This could be related to the fact that larger actors are generally more likely to win a contest with other group members [76–83] since size generally correlates with strength and the ability to inflict injury [84]. Therefore, smaller actors can only be more aggressive if they have an outside option available, as they experience higher probabilities of being evicted from the group by their larger counterparts [33]. Additionally, conflict generally increased as actors became larger than receivers, regardless of outside options. In other words, larger actors were more aggressive when the recipient was much smaller than them. This finding is contrary to general expectations that conflict is heightened between similar-sized individuals within size-based hierarchies [33]. However, *D. aruanus* individuals do not form size hierarchies with strict size ratios and a queuing inheritance system [45]. As such, the incentive for elevated conflict between similar-sized individuals, as seen in other social fishes with limited mobility and strict queuing systems, is reduced. Instead, individuals may have targeted aggression towards much smaller group members as they may represent lower quality potential mates or as a means of limiting group size—thereby, resulting in increased conflict as actor: recipient size ratios increased.

Taken on balance, these results suggest that in *D. aruanus*, large individuals and individuals of similar size engage in less conflict when ecological constraints are relaxed and hence the presence of a vacant coral can affect the intensity of competitive interactions in certain ecological and social contexts.

### (b) Cooperation and ecological constraints

In general, the ecological constraints hypothesis predicts that cooperation will decrease in groups as ecological constraints are relaxed, and the magnitude of the effect will be modulated by individual traits, social context and ecological context [1]. The basic rationale is that individuals will be less willing to engage in cooperation within the group as their outside options improve. On the contrary, our results suggest that the intensity of peacefully cooperative interactions (i.e. submissive and affiliative behaviours) was unrelated to ecological constraints and the other social and ecological factors measured in this study, except for individuals in larger groups, which were generally more submissive. A possible explanation for these results could be that the duration of the video recording (30 minutes) was not enough for measuring a change in the frequency of peacefully cooperative interactions in response to our experimental manipulation. It is well-known that peacefully cooperative interactions can be a response to previously received conflictive interactions—subordinates use both submissive and affiliative behaviours to mitigate dominant aggression and increase their likelihood of being tolerated within their group [53,85–87]. In a context where both cooperation and conflict are occurring, as in *D. aruanus,* conflictive interactions directed from dominants towards subordinates probably represent the first observable response to any experimental manipulation, such as a relaxation of ecological constraints. Subordinate individuals may therefore need some time to properly adjust their behaviour (in the form of peacefully cooperative interactions) in response to any change in within-group conflict, and this possibility would require further testing in future studies.

## 5. Conclusion

Our work investigated the effect of the manipulation of ecological constraints on both within-group cooperation and conflict in complex coral reef fish societies by measuring a range of different ecological and social factors. The results of this study suggest that to truly understand how conflict and cooperation respond to ecological constraints, it is imperative to incorporate variation in individual body size, size differences, territory size, group size, habitat availability, conspecific density and intergroup interactions into theoretical models. To date, our general understanding of how different ecological contexts influence cooperation and conflict between individuals is still in progress, and further experimental manipulations and comparative analyses across different systems and taxa would be helpful in shedding further insights. Additionally, our results highlight the need to refine existing models of social evolution to better incorporate ecological and social heterogeneity. By linking experimental manipulation to natural variation in group and habitat characteristics, this study contributes to a more nuanced understanding of how ecological constraints influence dispersal decisions, conflict and group stability in animal societies.

## Supplementary Material



## Data Availability

Data, code and README uploaded as supplementary material. Supplementary material is available online [88].
