# Supplementary material for: Experimental manipulation of ecological constraints influences conflict but not cooperation in the humbug damselfish Dascyllus aruanus
Source: Proc Biol Sci. 2026 Oct 14;293(2080):20253229. doi: 10.1098/rspb.2025.3229 (PMC13678518; doi:10.1098/rspb.2025.3229)
Supplement: Supplementary file 1 [file rspb.2025.3229.zip › rspb.2025.3229/rspb20253229_si_001.docx]

**Supplementary material**

**Experimental manipulation of ecological constraints influences conflict but not cooperation in the humbug damselfish *Dascyllus aruanus***

Branconi, R. ^1,2,3^, Wong, M. ^4^, Stouffer, D.B.^5^, Rueger, T.^6^ & Buston, P.M^1^.

1. Boston University, Department of Biology, 5 Cummington Mall, Boston, Massachusetts 02215; Email: branconi@bu.edu; buston@bu.edu.

2. ZARATAN: Marine Research & Higher Education Center - World Biodiversity Association – Sezione Arcipelago Toscano, Portoferraio, 57037 Italy; Email: rebecca.branconi@biodiversityassociation.org

3. The Pennsylvania State University, Department of Biology, 208 Mueller Laboratory, University Park, PA 16802-5301; Email: rqb5831@psu.edu

4. School of Earth, Atmospheric and Life, University of Wollongong, Wollongong, NSW 2522, Australia; Email: marianw@uow.edu.au.

5. [Leibniz Institute of Freshwater Ecology and Inland Fisheries](https://scholar.google.co.nz/citations?view_op=view_org&hl=en&org=16026954720564674820). Email: daniel.stouffer@igb-berlin.de

6. Dove Marine Laboratory, School of Natural and Environmental Sciences, Newcastle University, Newcastle Upon Tyne, UK. Email: [theresa.rueger@newcastle.ac.uk](mailto:theresa.rueger@newcastle.ac.uk).

**Published in *Proceedings of the Royal Society B: Biological Sciences:***

**https://doi.org/10.1098/rspb. XXX.XXX**

**Table S1.** Type II analysis of variance table for our full conflictive model showing effects of ecological or social factors. Log-likelihood test statistic (χ^2^), degrees of freedom (DF) and p-values of all fixed effects of the full generalized linear mixed model with gamma error term, using conflictive interactions as response variable. All numeric variables were scaled to improve model convergence. Group ID, actor ID and receiver ID were used as crossed random intercepts. Marginal R^2^ = 0.061, conditional R^2^ = 0.539.

**Table S2. a1)** Type II analysis of variance table for our full cooperative affiliative model showing no effect of ecological or social factors. Log-likelihood test statistic (χ^2^), degrees of freedom (DF) and p-values of all fixed effects of the full generalized linear mixed model with gamma error term, using peaceful cooperative affiliative interactions as response variable. All numeric variables were scaled to improve model convergence. Group ID, actor ID and receiver ID were used as crossed random intercepts. Marginal R^2^ = 0.133, conditional R^2^ = 0.389. **a2)** Type II analysis of variance table for our full cooperative submissive model showing no effect of ecological or social factors, with the exception of group size. Log-likelihood test statistic (χ^2^), degrees of freedom (DF) and p-values of all fixed effects of the full generalized linear mixed model with gamma error term, using peaceful cooperative submissive interactions as response variable. All numeric variables were scaled to improve model convergence. Group ID, actor ID and receiver ID were used as crossed random intercepts. Marginal R^2^ = 0.113, conditional R^2^ = 0.222. **b1)** Type II analysis of variance table for our reduced cooperative affiliative model with no two-way interaction showing no effect of ecological or social factors. Marginal R^2^ = 0.126, conditional R^2^ = 0.370. **b2)** Type II analysis of variance table for our reduced cooperative submissive model with no two-way interaction showing no effect of ecological or social factors, with the exception of group size. Marginal R^2^ = 0.106, conditional R^2^ = 0.215.
